# Christiaan Neethling Barnard — A controversial figure: Courage, innovation, and the global impact of the world’s first human heart transplant

**DOI:** 10.1590/1516-3180.2026.3619.16122025

**Published:** 2026-04-10

**Authors:** Noedir Antônio Groppo Stolf

**Affiliations:** I Faculty of Medicine, Universidade de São Paulo (USP), São Paulo (SP), Brazil.

In the early hours of May 26, 1968, Professor Euryclides de Jesus Zerbini and his associates performed the first human heart transplantation in Brazil. Years prior, the possibility of such operation had been proposed to him twice; however, he had considered it premature.

In December 3, 1967, Dr. Christiaan Neethling Barnard performed the first human-to-human heart transplantation worldwide. The following day, Dr. Zerbini began a series of meetings to prepare his team. As in several centers worldwide, the Brazilian team decided to pursue transplantation inspired by Barnard’s achievement.^1^

Dr. Barnard was born in November 8, 1922, in the rural town of Beaufort West, South Africa, approximately 300 miles from Cape Town. He later moved to Cape Town to study Medicine at the University of Cape Town and worked in other towns before returning to Groote Schuur Hospital (GSH).^2^

He was later awarded a scholarship to the University of Minnesota in Minneapolis, United States, under the leadership of the renowned Professor Owen Wangensteen. There, he observed the pioneering use of cardiopulmonary bypass by Walton Lillehei and Richard Varco.

After 30 months in the United States, most of that time spent far from his family, as he was married to a nurse and had two young children, Dr. Barnard returned to Cape Town. He brought a heart-lung machine funded by a grant from the U.S. National Institutes of Health (NIH), and immediately initiated an open-heart surgery program at GSH.

In the early 1960s, he considered heart transplantation. In January 1964, Dr. James Hardy performed the first cardiac xenotransplantation in a human using a chimpanzee heart.^3^ In addition to performing orthotopic heart transplants in dogs with his younger brother, Dr. Marius Barnard. He also traveled to the United States for 3 months to work with Dr. David Hume in Richmond, Virginia, gaining experience in immunosuppression for kidney transplantation. Coincidentally, Dr. Richard Lower recently transferred from Stanford to the same service, where he began an experimental heart transplantation program similar to that led by Dr. Norman Shumway at Stanford.

By late 1967, three American surgeons had announced their readiness to perform a heart transplant, or nearly attempted one: Dr. Shumway at Stanford, Dr. Lower in Richmond, and Dr. Adrian Kantrowitz at Maimonides Hospital in New York City.

After returning from Richmond, Dr. Barnard and Professor Velva Schrire, Chief of Cardiology at the GSH, selected the candidates for transplantation. Due to concerns that performing the first transplant in a non-white recipient during the apartheid era could be misinterpreted as experimenting on non-white patients, they decided that both the donor and recipient must be Caucasian. The recipient was a 53-year-old man with ischemic cardiomyopathy.

On the afternoon of Saturday, December 2, 1967, a 25-year-old woman, named Denise Darvall, was struck by a drinker and brought to GSH. After brain death was confirmed, her father consented to the donation of her heart and kidneys.

The transplantation was performed with the donor and recipient in adjacent operating rooms. As criteria for brain death had not yet been established, the team waited for the donor heart to stop beating before perfusing it via the heart-lung machine.

At the end of the procedure, after removing the aortic cross-clamp, Dr. Barnard attempted twice to wean the patient off the cardiopulmonary bypass, succeeding on the third attempt. The operation concluded at 6:15 a.m. on December 3, 1967.^2^

In the three Brazilian transplants Dr. Zerbini performed between 1968 and 1969, the heart was similarly perfused after circulatory arrest by inserting a cannula into the innominate artery and connecting it to a heart-lung machine.

Today, the original GSH building houses the Museum of the First Heart Transplant. The museum features a complete reconstruction of the operating rooms, Washkansky’s recovery room, and Dr. Barnard’s office, with numerous fascinating historical photographs.

A major early controversy arose after the surgery. As three American surgeons were on the verge of performing the first transplant (Shumway, Lower, and Kantrowitz), some accused Barnard of being reckless or insufficiently prepared. However, solid evidence showed that he had mastered surgical techniques in dogs and was trained in immunosuppression in Richmond. Ultimately, Barnard dared.^1,2^

Although Washkansky survived only 18 days, Barnard’s achievements reverberated worldwide. His second patient, Philip Blaiberg, survived 19 months.

Barnard immediately became an international celebrity. He was received by the Mayor of New York City and appeared on American television with Adrian Kantrowitz and Michael DeBakey. He also traveled to Europe, where he met Sophia Loren and later claimed to have had an affair with actor Gina Lollobrigida.^1^

After divorcing his first wife, he married two younger, wealthy women, fathering two children with each of his three wives. Although cardiovascular surgery at GSH continued under his associates, including his brother Marius Barnard, constant international engagements increasingly removed him from daily clinical practice, and his personal life became a target of criticism.

Despite these developments, the challenges of early graft failure and severe rejection in the pre-cyclosporine era prompted Barnard and his junior Belgian colleague, Jacques Losman, to develop a heterotopic heart transplantation model in 1975. Although rarely used today, this technique played an important role.

Barnard retired early from GSH at age 61, citing arthritis. He has dedicated himself to public speaking, writing, and controversial involvement with a cosmetics company.

In June 2000, Prof. Alain Carpentier organized a congress in Paris, inviting all surviving pioneers and a group of “grand invités.” During closing dinner at the Eiffel Tower, as I was looking for a table with a good view of Paris, someone waved at me. Upon approaching, I barely recognized Dr. Barnard, who had undergone nasal graft reconstruction following cancer. He told me, ‘I am living in the best city in the world, Vienna, and taking care of the Christian Barnard Foundation.’ The patient died on September 2, 2001, from an asthma attack while swimming in a hotel pool in Paphos, Cyprus.

How will Christiaan Barnard be remembered in retrospect? He will be remembered as the surgeon who had the courage to perform the world’s first human heart transplant. To avoid criticism, he insisted on waiting for the donor heart to stop beating, a decision later introduced heterotopic heart transplantation, an innovative technique well-suited to the pre-cyclosporine era. Unfortunately, his personal life and public behavior led some colleagues to view him as a “playboy.”

Therefore, in my opinion, these aspects should not detract from the contributions of this fascinating and charismatic individual, whom I had the privilege of meeting.
